# Disseminated Tumor Cells (DTCs) in Patients with Cervical Cancer Reveal Mesenchymal Properties and Potential Therapeutic Targets—A New Perspective?

**DOI:** 10.3390/ijms27114875

**Published:** 2026-05-28

**Authors:** Elisa Brochwitz, Ivonne Nel, Anne Eckardt, Laura Weydandt, Anne Kathrin Höhn, Karsten Winter, Bahriye Aktas

**Affiliations:** 1 Department of Gynecology, Medical Center, University of Leipzig, 04103 Leipzig, Germany; 2 Department of Pathology, Medical Center, University of Leipzig, 04103 Leipzig, Germany; 3 Institute of Anatomy, Medical Faculty, Leipzig University, 04103 Leipzig, Germany

**Keywords:** cervical cancer, disseminated tumor cells (DTCs), circulating tumor cells (CTCs), phenotype, Vimentin, cytokeratin, epithelial–mesenchymal transition (EMT), PD-L1, VEGF, p16

## Abstract

Despite effective primary treatment, approximately 20% of cervical cancer patients experience recurrence, which may be driven by early hematogenous dissemination. While circulating (CTCs) and disseminated tumor cells (DTCs) are established prognostic markers in breast cancer, their role in cervical cancer remains poorly defined. Current detection methods rely predominantly on cytokeratin (CK) expression, but epithelial–mesenchymal transition (EMT) may lead to CK downregulation, potentially compromising detection sensitivity. We analyzed bone marrow (*n* = 43) and blood samples (collected pre-surgically and at follow-up) from cervical cancer patients. DTCs and CTCs were detected using a standardized CK based immunocytochemical assay. A multi-parameter immunofluorescence (IF) approach was employed to simultaneously assess CK, vimentin (Vim), p16, PD-L1 and VEGF on DTCs. Marker expression profiles were compared with matched tumor tissue and correlated with clinical and pathological parameters. CTCs were detected in 16% (7/43) of pre-surgical blood samples. DTC positivity increased from 56% (24/43) using CK based detection to 74% (32/43) when assessed via multi-parameter IF. A total of 248 DTCs were identified (median: 7 per patient). The most prevalent DTC phenotype was Vim+ (54%), followed by CK+ (12%), Vim+/VEGF+/p16+ (7%), and CK+/Vim+ (4%). Significant correlations were observed between p16 and VEGF (r = 0.631, *p* < 0.001) and between PD-L1 (r = 0.323, *p* < 0.001). Discordance between DTCs and tumor tissue reached 43–64%, primarily attributable to PD-L1 gain in DTCs. Over 70% of DTCs lacked CK, with majority exhibiting vimentin positivity, indicative of a mesenchymal phenotype. Vim+ and VEGF+ DTCs were associated with disease recurrence, suggesting their prognostic value. These findings underscore the limitations of relying solely on CK for DTC detection and highlight the potential of EMT and immune-related markers as novel biomarkers in cervical cancer. Further validation in larger, prospective cohorts is warranted.

## 1. Introduction

Despite advancements in screening and prevention, cervical cancer remains a significant global health concern. With approximately 660,000 new cases and 350,000 deaths annually, it ranks as the fourth most common malignancy in women worldwide [1]. In Germany, the incidence is markedly lower, largely due to widespread human papillomavirus (HPV) vaccination programs and routine cytological screening. Yet, about 1500 women still succumb to the disease each year, underscoring the persistent challenge of recurrence and late relapse [2].

The vast majority of cervical cancers are driven by persistent infection with high-risk HPV types, particularly HPV 16 and 18 [3]. Viral oncoproteins E6 and E7 interfere with tumor suppressor functions, leading to an overexpression of the cyclin-dependent kinase inhibitor p16, promoting genomic instability and carcinogenesis [4,5]. While early-stage disease is often curable with surgery or chemoradiotherapy, a subset of patients experience disease recurrence or distant metastases, sometimes years after initial treatment. One proposed mechanism is the early hematogenous dissemination of tumor cells which may seed distant niches such as the bone marrow [6].

These disseminated tumor cells (DTCs) are exceedingly rare events and may persist in a dormant state, evading detection until reactivation leads to clinical relapse or overt metastasis [7]. In early-stage breast cancer, DTCs in the bone marrow are well studied and have been established as independent prognostic markers with potential implications for risk stratification and therapeutic targeting [8,9,10]. Similarly, circulating tumor cells (CTCs) in the blood have established clinical utility as dynamic prognostic biomarkers in metastatic breast cancer [11]. In cervical cancer, however, data concerning DTCs and CTCs remain sparse and inconsistent. Reported DTC detection rates vary widely from 16% to 29%, and their clinical relevance is still under investigation [12,13,14,15,16]. Only a few studies analyzed CTCs, and detection rates are even more variable, ranging between 26% and 81% [17,18].

In this study, we investigated DTCs and CTCs from 43 cervical cancer patients using the standardized concept adapted from breast cancer protocols [12] which is based on the epithelial marker cytokeratin (CK). However, during the process of epithelial–mesenchymal transition (EMT), tumor cells may downregulate epithelial markers such as CK while acquiring mesenchymal traits, including increased motility, invasiveness, and immune evasion [19,20,21,22]. Consequently, DTCs with mesenchymal or hybrid features may be missed by conventional CK based assays. To address this limitation, we employed an additional sequential multi-parameter immunofluorescence (IF) strategy to comprehensively characterize epithelial and non-epithelial DTCs. We further assessed the expression of p16, VEGF and PD-L1 on DTCs to explore their potential prognostic and therapeutic relevance. Finally, we compared marker expression on DTCs with matched tumor tissue collected at diagnosis to evaluate intra-tumoral heterogeneity and dynamic changes during disease dissemination.

## 2. Results

### 2.1. DTC and CTC Status Using a Standard Brightfield Method Based on Pan-CK

**Figure 1:**
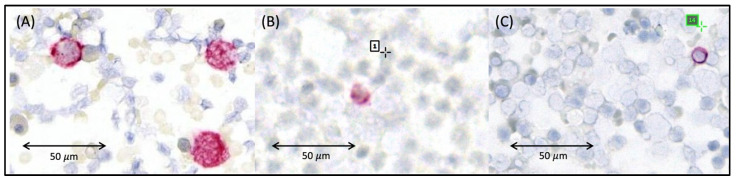
Representative microscopic images of Cytokeratin (CK) positive tumor cells. (**A**) Reference Cell (HCT116) from a control slide. (**B**) Patient-derived circulating tumor cell (CTC) which stained positive for CK and (**C**) disseminated tumor cell (DTC) from the same patient that stainend positive against CK. CK-positive cells are visualized in pink, while nuclei are counterstained in blue. Images were processed and annotated using Aperio ImageScope software Version 12.4.6 (Leica Biosystems, Nußloch, Germany) and the figure was assembled using Microsoft PowerPoint.

Using the standardized DTC detection method that is usually applied to bone marrow samples from breast cancer patients [12,23], we analyzed bone marrow aspirates of 43 patients with cervical cancer, including 40 patients with a primary tumor and three patients with recurrenct disease at baseline. Additionally, we assessed the corresponding pre-surgical blood samples that were collected one day before oncologic surgery. With the CK based detection method we found DTCs in 24/43 cases (56%). CTCs were identified in seven of the 43 pre-surgical blood samples (16%) whith 36 patients (84%) being CTC negative at baseline. The CK based method further revealed that among the CTC positive cases, one patient (ID: 2063) was DTC negative in the bone marrow, while six were positive for epithelial DTCs. Of the CTC negative patients, 18 (49%) revealed a positive DTC status, while 18 were DTC negative (49%) and in one case the bone marrow sample was missing. During clinical follow-up visits, 39 of the 43 patients provided repeated blood samples. Among the seven initially CTC positive patients, one (ID: 2063) remained CTC positivethroughout follow-up, despite being negative for CK+ DTCs, while four became CTC negative within 3–7 months post-surgery. In two cases, follow-up blood samples were unavailable. The latter six revealed a positive DTC status using the CK based brightfield detection method, though (Figure 1).

### 2.2. Staining of Reference Cells Using Sequential Immunofluorescence

**Figure 2:**
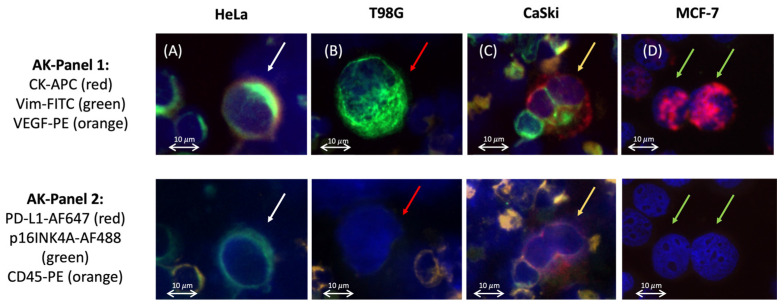
Positive and negative control cells for both marker panels. (**A**) HeLa (white arrows): in the first panel positive for cytokeratin (CK), Vimentin (Vim), VEGF and in the second panel positive for p16INK4A, negative for PD-L1 and CD45. (**B**) T98G (red arrows): positive for Vim. (**C**) CaSki (yellow arrows): positive for CK, Vim, VEGF, PD-L1 and p16INK4A. (**D**) MCF-7 (green arrows): positive for CK. All cell lines were negative for CD45. Only hematopoietic cells showed CD45 positive staining. Scale bar = 10 μm. Images were processed using Zeiss ZEN 3.7 software and the figure was assembled using Microsoft PowerPoint.

For the DTC subtype analysis, a multi-parameter immunofluorescence (IF) approach was essential to simultaneously identify epithelial, mesenchymal, HPV-associated and therapeutically relevant markers. To this end, we adapted our previously published sequential IF staining protocol which was originally developed for breast cancer samples [23,24] for the detection and phenotypic characterization of DTCs in cervical cancer patients. Positive and negative controls were included in every experiment. We used the MCF-7 breast cancer cells, along with a mixture of bone marrow cells spiked with cervical cancer cell lines (CaSki and HeLa) and the T98G glioblastoma cell line (Figure 2 and Table 1).

### 2.3. Multi-Parameter DTC-Detection and Quantification in Patient-Derived Samples

**Figure 3:**
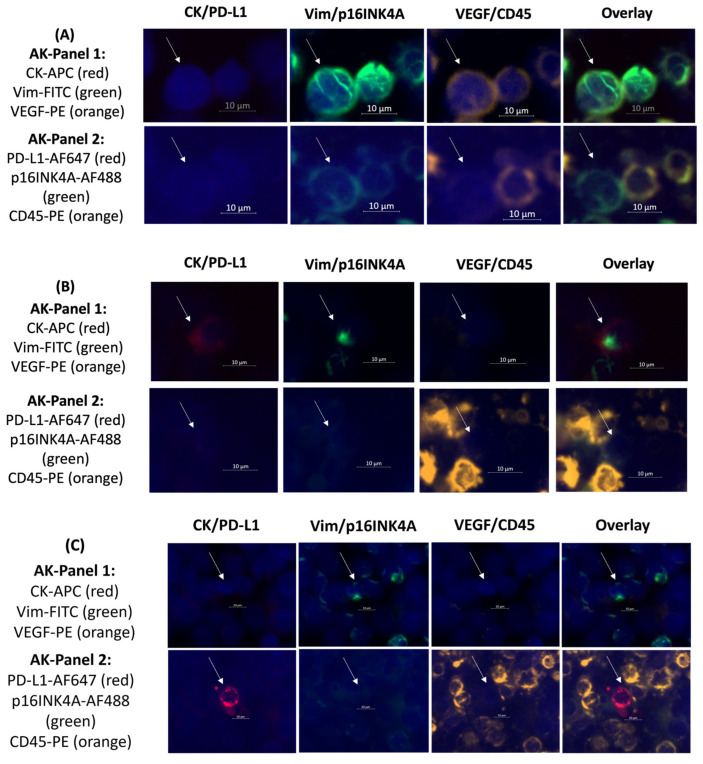
Representative images of patient-derived DTCs with three different profiles. (**A**) DTC positive for Vim, VEGF and p16, negative for CK, PD-L1 and CD45. (**B**) CK and Vim positive while negative for VEGF, PD-L1, p16 and CD45. (**C**) DTC positive for Vim and PD-L1, negative for all other markers. Scale bar = 10 μm. Images were processed using Zeiss ZEN 3.7 software and figure was assembled using Microsoft PowerPoint.

To identify DTCs in bone marrow samples from patients with cervical cancer, we applied combined morphologic and immunophenotypic criteria. Tumor cells were defined by an increased nucleus-to-cytoplasm ratio, positive nuclear staining with DAPI, absence of CD45 expression to exclude hematopoietic cells, and positive IF staining for at least one of the following markers: Pan-CK, Vim, VEGF, PD-L1 or p16INK4A—either individually or in combination. If the pre-defined morphologic and immunphenotypic criteria were met, a cell was captured and counted as a DTC. Diffuse or non-cell-associated fluorescent signals were excluded from analysis to minimize false-positive classification. Unlike the scoring system in immunohistochemical staining using tissue, and similar to previous work [23,24], the fluorescent signal of a DTC was evaluated either positive or negative to assess markers such as PD-L1 or VEGF. This approach enabled consistent, objective characterization of DTC phenotypes across the cohort. Representative images of patient-derived DTCs with different profiles are shown in Figure 3.

**Figure 4:**
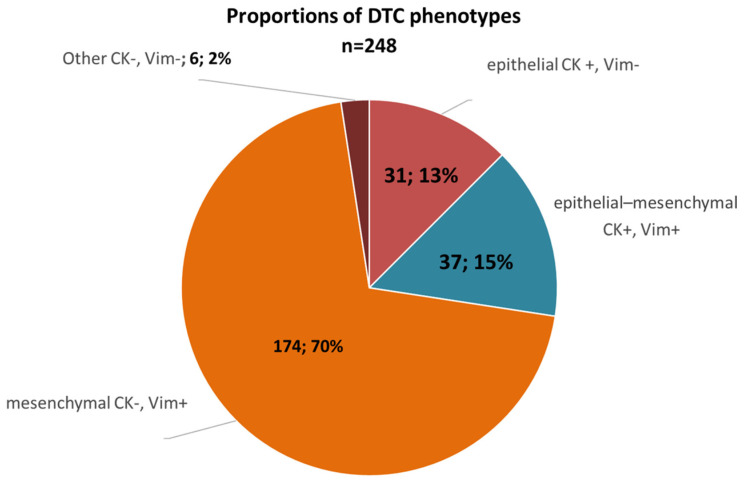
Proportion of DTC Phenotypes. The majority of detected DTCs (70%, *n* = 174) was positive for Vimentin without CK expression, hence revealing a mesenchymal phenotype. An epithelial phenotype was found in 13% (*n* = 31) of the DTCs and 15% (*n* = 37) presented epithelial–mesenchymal transition (EMT)-like properties. Chart generated using Microsoft Excel.

Using the multi-parameter detection approach, we detected 248 DTCs among the cohort (Table 2). Of the 43 cervical cancer patients, 32 (74%) revealed a positive DTC status and we detected a median number of 7 DTCs per patient (ranging from 1 to 17 DTCs). Investigating the phenotype, 13% (*n* = 31) of the DTCs were epithelial, 15% (*n* = 37) displayed EMT-like features and 70% (*n* = 174) revealed a mesenchymal phenotype while 2% were classified as other (Figure 4 and Table 2).

In total, we found that 68 of the DTCs were CK positive (CK+, 27.4%) and 180 were CK negative (CK−, 73%) while 211 DTCs revealed Vim (85%). A subpopulation of 138 DTCs was positive for Vim only (Vim+, 55%) and 174 DTCs (70%) revealed Vim together with other markers such as VEGF.

Subsequently, the markers were distributed among the detected DTCs as follows: 85% Vim+, 27% CK+, 21% p16+, 20% VEGF+ and 11% PD-L1+. Spearman Rho correlation showed that the presence of Vim on a cell was strongly linked to the lack of CK (r = −0.529, *p* ≤ 0.001). Further the expression of p16 was strongly correlated to VEGF (r = 0.631, *p* ≤ 0.001) and moderately correlated to PD-L1 (r = 0.323, *p* ≤ 0.001).

**Figure 5:**
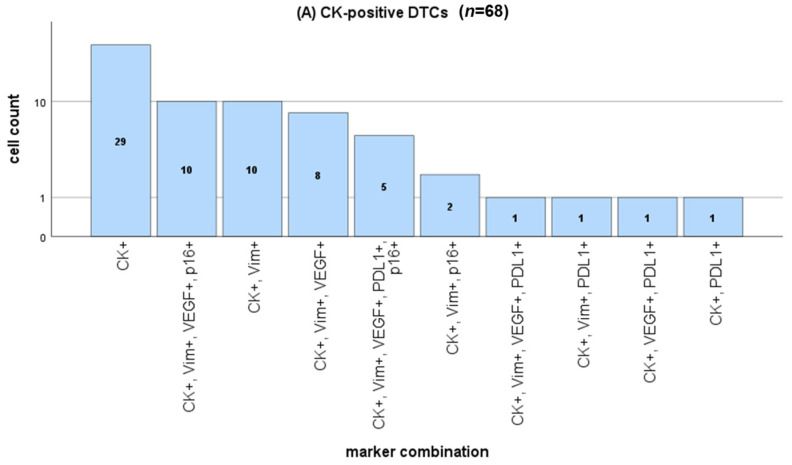

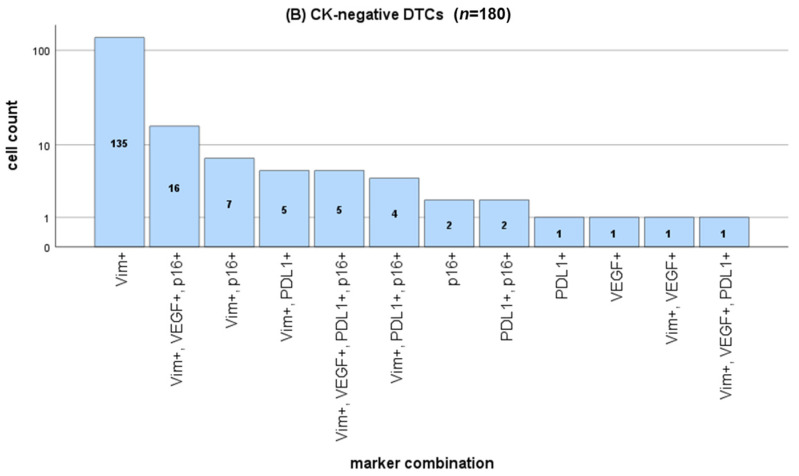
DTC quantification in (**A**) CK positive subpopulations and (**B**) CK negative subpopulations. Statistical graphs were generated using IBM SPSS Statistics Version 29.0.

In total, we were able to identify 22 DTC subpopulations which were distinguished into CK positive and CK negative DTCs. The CK+ DTCs (*n* = 68) can be divided into 10 different subpopulations (Figure 5A) with CK+ being the most frequently occurring profile (*n* = 29) followed by CK+Vim+VEGF+P16+ (*n* = 10) and CK+Vim+ (*n* = 10). The majority of detected DTCs belonged into the CK− category (*n* = 180) consisting of 12 subpopulations (Figure 5B). The most frequently presented feature among CK− DTCs was the expression of Vim (*n* = 135) followed by Vim+VEGF+p16+ (*n* = 16) and Vim+p16+ (*n* = 7).

### 2.4. Comparison of p16, VEGF and PD-L1 in Matching Tumor Tissue

**Figure 6:**
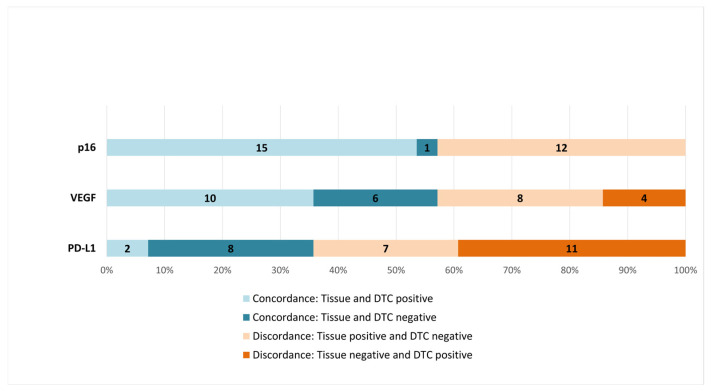
Comparison of p16, VEGF and PD-L1 in matching tumor tissue. In four cases we found VEGF on DTCs but not in matching tumor tissue, indicating a potential therapeutic target. Further, we detected PD-L1 on DTCs in 11 patients that had PD-L1 negative tissue at diagnosis, suggesting a potential discordance between tumor tissue and DTC phenotype. Chart generated using Microsoft Excel.

Matching tumor tissue gained at diagnosis was available in 28 of 32 DTC positive cases. We compared the p16, VEGF and PD-L1 expression in tumor tissue vs. DTCs and found discordance rates ranging from 43% to 64% (Figure 6). In more detail, PD-L1 discordance was observed in 64% of the cases with 11 patients showing a positive PD-L1 staining on DTCs but not in tumor tissue. Vice versa, seven patients showed PD-L1 positive staining in the tumor tissue but were PD-L1 negative on DTCs. Further, PD-L1 was concordant in 36% of the cases with eight patients being PD-L1 negative and two patients being PD-L1 positive in both specimens.

VEGF was discordant in 43% with four patients showing VEGF positive DTCs but negative tissue staining suggesting a VEGF gain and eight patients with VEGF negative DTCs but positive tissue. VEGF concordance was found in 57% of the patients, with 10 positive and six negative cases. Interestingly, p16 was also discordant in 43%, but only in the form of loss. Twelve patients showed a positive p16 staining in tissue, but not on DTCs. As expected, there were no p16 positive DTCs in patients with p16 negative tissue. The concordance rate was 57%, meaning that 15 patients were p16 positive and one was negative.

### 2.5. Correlation of DTC Subtypes to Clinical Parameters

A positive DTC status was correlated to lymphovascular invasion (r = 0.362, *p* = 0.017) which itself was associated with tumor stage (r = 0.594, *p* < 0.001) and menopausal status (r = 0.487, *p* = 0.005). Tumor stage was also correlated with the presence of CK− (r = 0.358, *p* = 0.019) and Vim+ DTCs (r = 0.400, *p* = 0.008). At the time of bone marrow aspiration, three patients were diagnosed with recurrent disease (Table 1. Patient Characteristics). Mann–Whitney U testing showed that the presence of VEGF+ DTCs was significantly associated with recurrent disease (*p* = 0.04) which presented an increased mean number compared to patients sampled at primary diagnosis (mean VEGF+ DTCs: 3.33 vs. 1.3; *n* = 3 vs. 29). During the follow-up, three additional patients developed recurrence. In these cases, Mann–Whitney U testing showed significantly increased mean numbers of Vim+ DTCs (*p* = 0.024) respective CK− DTCs (*p* = 0.009) compared to non-recurrent patients (Vim+: 10.5 vs. 3.0 and CK−: 9.0 vs. 3.0). Among all six patients who experienced recurrence, either at baseline or during follow-up, we found a significantly higher median DTC count compared to patients with primary tumors (11.0 vs. 6.0, *p* = 0.001). CK− DTCs were significantly more frequent in recurrent cases (*p* = 0.001) as were VEGF+ DTCs (*p* = 0.033) and Vim+ DTCs (*p* = 0.006). Notably, all patients who developed recurrence harbored Vim+ DTCs. Given the small number of recurrent cases (*n* = 6), all statistical associations are reported with appropriate caution and should be interpreted as exploratory. Two patients were diagnosed with metastatic disease and Spearman Rho test showed a significant correlation to PD-L1+ DTCs (r = 0.383, *p* = 0.03). Interestingly, the median number of p16+ DTCs was inversely correlated with the menopausal status of the patients (r = −0.574, *p* < 0.001), indicating that increasing age and menopause were associated with the loss of p16 on DTCs. Consequently, menopausal status was inversely correlated with the detection of Vim+VEGF+p16+ DTCs (r = −0.385, *p* = 0.03) but positively correlated with CK+Vim+VEGF+ DTCs (r = 0.467, *p* = 0.007).

### 2.6. Multi-Parameter Immunofluorescence vs. Conventional CK Based Immunocytochemical DTC Detection

**Figure 7:**
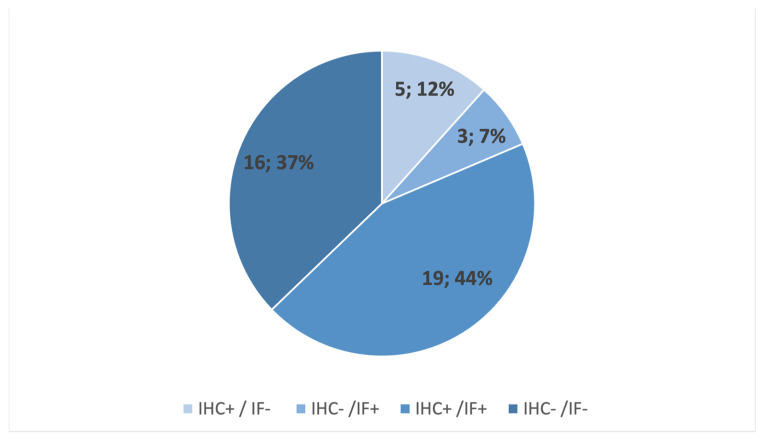
DTC Status based on CK detected with immunocytochemistry (ICC) vs. IF. Of the patients which presented CK+ DTCs 44% (*n* = 19) were positive using both methods and 37% (*n* = 16) were DTC negative in both methods. Chart generated using Microsoft Excel.

Our multi-marker approach revealed a DTC positivity rate of 74% (32/43 patients) including 51% (22/43 patients) presenting CK+ DTCs. Using the conventional CK based detection method that is commonly used for breast cancer patients, the positivity rate was 56% (24/43 patients). Among the cohort, 12% (*n* = 5) were only DTC positive using the conventional (CK based) brightfield method and 7% (*n* = 3) were only CK+ DTC positive using the fluorescent staining approach (Figure 7). Looking exclusively at the patients which revealed CK+ DTCs using both methods, 44% (*n* = 19) had a positive DTCs status and 37% (*n* = 16) were DTC negative resulting in a comparability of 81% (35/43) for the epithelial DTC status.

Using the additional markers Vim, PD-L1, p16 and VEGF lead to an increased DTC detection rate. Consequently 74% (*n* = 32) were DTC positive and only 26% (*n* = 11) were DTC negative. In eight cases we found DTCs only with the fluorescent multi-marker method but not when applying the brightfield detection, although three of them had CK+ DTCs (Table 3). Altogether, Spearman Rho correlation revealed that both methods are comparable in terms of DTC status (r = 0.659; *p* < 0.001). But looking at the phenotype and potential targets draws a different picture.

## 3. Discussion

In our study, we showed that CTC detection based on epithelial markers such as CK leads to relatively low detection rates in patients with cervical cancer. Only 16% of all 43 cases revealed a positive CTC status using the standardized method initially designed for DTC detection in breast cancer. For comparison, a study based on CK combined with conditionally replicative adenovirus targeting telomerase-positive cells was performed by Takakura et al. and reported 26% CTC positivity (in six of the 23 tested patients) [17]. Tewari et al. analyzed blood samples of 176 patients with advanced cervical cancer using an EpCAM-dependent enrichment approach followed by staining against CK. They found CTCs in nearly every case prior to and in 81% after one cycle of therapy [18]. Possible reasons for the low CTC detection rate in our study might be the rare incidence of epithelial CTCs in patients with low tumor stages and technical issues such as high cell density on microscopic slides and undesired background staining. Notably, Du et al. investigated CTCs in blood of 107 patients with cervical cancer using negative selection via depleting CD45+ hematopoietic cells followed by fluorescence in situ hybridization (FISH) with CEP8 in remaining cells to find non-hematopoietic cells. They reported a CTC positivity rate as high as 80% with counts ranging from 0 to 27 CTCs per 3.2 mL blood [25]. Another CK independent approach was recently described by Reginacova and colleagues who employed a size-based filtration method followed by qPCR of tumor-associated genes to detect CTCs in 30 patients with cervical cancer. They found CTCs in 96.5% of the tested patients [26].

In contrast to the few existing studies which investigated either CTCs or DTCs, we looked at both. Our CK based detection method revealed that half of the CTC negative patients had a positive DTC status. The overall CK based DTC positivity was 56% in our cohort which is higher compared to other publications and indicates potential to identify patients with minimal residual disease (MRD) and hence elevated risk for recurrence even if CTC analysis is insufficient. For comparison, a study by Scheungraber et al. analyzed DTCs in 24 patients with HPV-positive cervical cancer using a HPV type-specific nested PCR enzyme immunoassay. They found that six patients (25%) were DTC positive [27]. In a cohort of 325 cervical cancer patients, Fehm et al. investigated DTCs using the standardized CK based method and reported a positivity rate of 22% ranging from 18% to 45% depending on the tumor stage with no prognostic relevance [13].

By applying a modified sequential multi-parameter DTC detection procedure which employs CK in combination with Vim, VEGF, PD-L1 and p16 we discovered that 74% of the patients had DTCs in the bone marrow at the time of oncologic surgery. Notably, we found 22 subpopulations among the 248 detected DTCs and revealed that the majority presented a mesenchymal phenotype. The most frequently detected profiles were Vim+/CK− (*n* = 135) followed by CK+ (*n* = 68) DTCs. Due to the lack of studies concerning non-epithelial DTCs in cervical cancer we can only compare our results to CTC studies. Hence, our data is in line with a study by Pan et al. who described the occurrence of mesenchymal CTCs (23%) next to epithelial CTCs (39%) and CTCs with mixed phenotypic features (14%) in a cohort of 90 patients with early cervical cancer. They found that mesenchymal phenotypes were related to lymph node metastasis and lymphatic vascular invasion [28]. Further, a study by Gies et al. analyzed phenotypic characteristics of CTCs in 115 patients with cervical cancer and postulated that the post-therapeutic detection of PD-L1-positive CTCs was associated with shorter recurrence-free survival [29].

In our study, we compared PD-L1, VEGF and p16 expression in DTC-positive cases with available matching tumor tissue (*n* = 28) and revealed additional potential therapeutic targets. In four cases we found VEGF and in 11 cases PD-L1 was expressed on DTCs but not in the tissue biopsy. In the literature, VEGF in tumor cells was described to be associated with promoted cell proliferation, survival, dedifferentiation, motility and maintenance of cancer stem cell properties [30]. Moreover, in tumor cells, VEGF might act beyond angiogenesis and vascularization. Autocrine and paracrine VEGF signaling could contribute to key aspects of carcinogenesis and hence be linked to therapy resistance and tumor progression [31]. In this context, bevacizumab, a monoclonal antibody against VEGF, has been successfully implemented in the treatment of advanced cervical cancer. The phase III GOG-240 trial demonstrated that the addition of bevacizumab to standard chemotherapy in patients with recurrent, persistent or metastatic cervical cancer significantly improved overall survival, thereby establishing VEGF inhibition as an effective therapeutic strategy in this setting [32]. The PD-L1 discordance (tumor negative, but DTC positive) might indicate a PD-L1 gain and hence promoted tumor immune escape as well as possible response to immune checkpoint inhibitors targeting the PD-1/PD-L1 axis [33,34]. This concept is further supported by the results of the KEYNOTE-826 trial, in which the addition of the immune checkpoint inhibitor pembrolizumab to standard chemotherapy with or without bevacizumab significantly improved overall and progression-free survival in patients with recurrent, persistent or metastatic cervical cancer, particularly in tumor expressing PD-L1. These findings highlight the clinical relevance of PD-L1 as a predictive biomarker and suggest that even subpopulations of tumor cells, such as PD-L1 positive DTCs, may contribute to treatment response to immune checkpoint inhibitors [35]. In contrast to VEGF and PD-L1, where expression was frequently gained in DTCs, p16 was predominantly lost in DTCs. Usually, increased p16 can contribute to cellular senescence and cell cycle arrest [36,37]. As p16 is a cyclin-dependent kinase (CDKN2A) inhibitor, it functions as a tumor suppressor by blocking CDK4/6 activity and hence arresting the cell cycle by hypophosphorylation of the retinoblastoma (Rb). Mechanistically, in cervical cancer a high-risk HPV infection is leading to Rb inactivation which causes a compensatory p16 overexpression [38]. We found 43% discordance between p16 expression in tumor tissue vs. DTCs suggesting that tumor cells might lose p16 on their way from the primary tumor site to the bone marrow. Correlation with clinical data showed that the p16 loss was associated with menopause and hence increasing age. More interestingly, VEGF+ DTCs were linked to recurrent disease and so were Vim+ and CK− DTCs.

Taken together, we revealed that both the brightfield and the multi-parameter method showed comparable DTC-positive rates based on CK (51% vs. 56%). The multi-parameter approach, however, increased the DTC rate up to 74% and revealed potential therapeutic targets that were not detected in tissue biopsies at diagnosis.

While our analysis relied on a binary classification approach for marker expression, the absolute number of DTCs may still provide valuable biological context. In our cohort, the median number of DTCs per patient was seven (range: 1–48), with a total of 248 DTCs identified. Notably, patients with recurrent disease had a higher median DTC count (11.0 vs. 6.0) compared to those with primary disease. Although the subcohort was very small, the mean number of Vim+ and VEGF+ DTCs was significantly higher in recurrent patients, indicating that the phenotypic profile of DTCs may be more informative than total count alone. These findings suggest that while DTC burden may reflect the extent of dissemination, the presence of aggressive phenotypes, particularly mesenchymal and immune-related markers, may be more directly linked to clinical progression.

As the majority of detected DTCs showed mesenchymal properties, our results raise the question whether CK is a suitable marker for DTC and CTC detection in patients with cervical cancer. For example, one of the pre-surgically CTC negative patients (ID 1446) was tested CK+ DTC negative using the CK based standard method. Using our multi-parameter approach, however, we found Vim+, p16+ and VEGF + DTCs which was in concordance to the tissue staining. Notably, this patient became CTC-positive during follow up and eventually developed recurrence. Another patient (ID 2063) was CTC-positive prior to surgery. We did not detect CK+ DTCs, but revealed p16+, VEGF+, Vim+ and PDL1+ DTCs. Noteworthy, the tumor tissue was negative for PD-L1, but positive for VEGF and p16. After radio-chemotherapy we detected persistent CTCs and hence she remained at increased risk for recurrence.

Further, one of the initially CTC-positive patients (ID 2837) was positive for CK+ DTCs. In addition, we found Vim+, PD-L1+ and VEGF+ DTCs which indicated a PD-L1 gain compared to the matching tissue sample. Although she became CTC-negative (CK based method), during follow up she developed recurrence. Since CTCs are rare events and the detection among millions of hematologic cells is challenging, it is likely that pre-surgical CTCs were simply overlooked. Another possibility is that CK+ CTCs were eliminated after surgery and adjuvant chemotherapy. As the mesenchymal properties of tumor cells are linked to resistance [39], multi-parameter staining should be applied for CTC analysis in future studies. Unfortunately, the collected blood volumes in our study were too small for the additional preparation of slides that could have been subjected to multi-parameter staining.

Although the cohort is too small to draw conclusions, the results add on to possible prognostic value of CK+ CTCs as well as mesenchymal DTCs.

## 4. Materials and Methods

### 4.1. Study Population and Informed Consent

**Figure 8:**
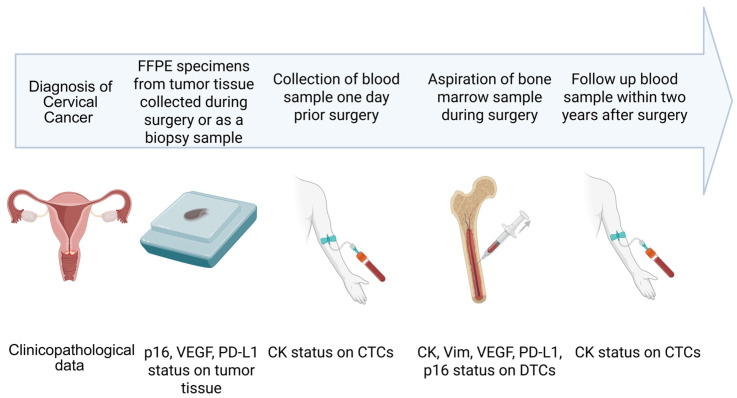
Study design. At diagnosis a tissue biopsy was obtained to histologically confirm cervical cancer cases. The FFPE tissue samples were stained against p16, PD-L1 and VEGF for comparison with DTC profiles. One day prior to oncologic surgery, blood samples were collected for CTC detection using the standardized CK based brightfield method. Bone marrow aspirates were sampled during surgery and DTCs were analyzed using the CK based and the multi-parameter IF staining method. Post-operative follow-up blood samples were collected within two years after surgery during routine visits if applicable. Clinical Outcome was assessed up to February 2025. Created with BioRender.com.

This research took place at the Department of Gynecology, University Hospital Leipzig, Germany. In accordance with the guidelines of our institutional ethics committee, patients diagnosed with histologically confirmed cervical cancer provided their written informed consent (internal reference number: No. 216/18-ek). It facilitated the collection of bone marrow and tissue samples during their surgical procedure or examination in anesthesia, as well as the collection of blood samples before and after the surgical procedure (Figure 8). Clinico-pathological data were retrieved from the patients’ medical records, and are detailed in Table 4. A total of 43 female patients that were treated in our clinic between 2021 and 2023 were enrolled in the study. The median age at sample withdrawal was 45, ranging from 30 to 73 years. Of the 43 included patients, 93% (*n* = 40) were diagnosed with primary cervical cancer at the time of bone marrow sampling, while 7% (*n* = 3) had a recurrent disease and all except for one case were HPV-associated carcinomas. During follow-up, 7.5% (*n* = 3) of the patients initially diagnosed with a primary tumor developed a recurrence. Two patients died from cervical cancer within the observation period. Cervical cancer management and subsequent follow-up evaluations were conducted within the setting of the Total Mesometrial Resection (TMMR) study at the Department of Gynecology, University of Leipzig [40]. Patients underwent either TMMR surgery, definite chemoradiotherapy, neoadjuvant or adjuvant chemotherapy in strict adherence to the latest treatment guidelines. The median follow-up time during our study period was 33.5 months (Range: 21–48 months).

### 4.2. Investigation of Blood Samples and Bone Marrow Aspirates

Per patient, 9 mL EDTA blood samples (S-Monovettes^®^, Sarstedt, Nümbrecht, Germany) were collected one day prior to surgery and during follow up. During oncologic surgery, 7–8 mL bone marrow samples were aspirated from both sides of the anterior iliac crest and immediately stabilized with heparin-sodium (ratiopharm, Ulm, Germany). Mononuclear cells were isolated by density gradient centrifugation using BioColl separation solution (Bio&Sell, Feucht, Germany) and transferred onto glass slides (1 × 10^6^ cells per slide) using a cytospin centrifuge (Tharmac, Limburg a.d. Lahn, Germany). Slides were fixed with ice-cold methanol and stored at 4 °C prior to further staining procedures. For DTC analysis, we prepared eight slides per bone marrow sample, with the remaining cell suspensions cryopreserved in liquid nitrogen for further investigation later on. For CTC analysis, two slides per blood sample were prepared. In total, bone marrow aspirates and blood samples from 43 patients were analyzed. We used a standardized brightfield detection method, utilizing an antibody directed against Pan-CK and labeled with alkaline phosphatase (Cat. No. 130-090-462, Miltenyi Biotech, Bergisch Gladbach, Germany). As a positive control with each run, we used reference slides with a defined mix of bone marrow cells and HCT116 cells. This protocol was based on previously established methods from our laboratory and was used to assess CK positive (CK+) DTCs on four of eight prepared bone marrow slides [23,24,41]. The same procedure was applied to analyze CK+ CTC. Both, DTCs and CTCs, were visualized in pink using alkaline phosphatase and short counterstaining with hematoxylin (Hollborn & Söhne, Leipzig, Germany) which colored the nuclei light blue. DTCs were semi-automatically detected and enumerated using the Aperio Versa microscope-based scanning system (Leica Biosystems, Nußloch, Germany) with rare events software that was trained to select DTC candidates according to color, shape, intensity and size while CTCs were counted manually according to predefined morphologic criteria [12,39].

Additionally, DTCs were analyzed using a novel sequential multi-parameter IF approach developed for this study, enabling phenotypic characterization beyond epithelial markers. Consequently, each bone marrow aspirate was assessed using both this innovative method and the established standard brightfield CK based detection technique.

### 4.3. Cell Culture for Reference Slides

For the standardized brightfield method, we used reference slides with a 50:1 mix of bone marrow cells and HCT116 colon carcinoma cells which were initially obtained from the American Tissue Culture Collection (ATCC). Cryo-preserved stock solutions were thawed and maintained under standard conditions (37 °C and 5% CO_2_ atmosphere) in Dulbecco’s Modified Eagle Medium (DMEM) containing 4.5 g/L glucose and l-glutamine (Cat. No. FG 0435, Biochrom, Berlin, Germany) supplemented with 10% fetal bovine serum (Cat. No. S 0615, Biochrom, Berlin, Germany) and 100 U/mL penicillin/streptomycin as published in previous work [23,24,41].

For the multi-parameter approach described in this study, the cervical cancer cell lines CaSki and HeLa, which exhibit a mixed epithelial–mesenchymal phenotype, were used alongside MCF-7 cells (epithelial breast cancer cell line) and T98G cells (mesenchymal-like glioblastoma cell line) to serve as positive and negative controls. This selection enabled validation of marker specificity across different tumor cell phenotypes. These cells were prepared, stored and fixed identically to our samples. They were initially obtained from the ATCC. For this study, aliquots were thawed and cells were cultured under standard conditions at 37 °C in an atmosphere of 95% air and 5% CO_2_. HeLa, MCF-7 and T98G cell lines were grown in DMEM containing 4.5 g/L glucose and l-glutamine (Cat. No. FG 0435, Biochrom, Berlin, Germany), while the CaSki cell line was cultured in Roswell Park Memorial Institute (RPMI) 1640 medium containing stable glutamine, 2.0 g/L NaHCO_3_ and phenol red (Cat. No. BS.FG1215, Bio&Sell, Feucht, Germany). Both media were supplemented with 10% fetal bovine serum (Cat. No.S 0615, Biochrom, Berlin, Germany) and 100 U/mL penicillin/streptomycin (PAN-Biotech, Aidenbach, Germany). They were harvested using trypsin/EDTA solution (PAN-Biotech, Aidenbach, Germany), re-suspended in culture medium, and incubated for 30 min to allow for cell surface recovery. After washing with phosphate-buffered saline (PBS), cell counts were performed using the Countstar (INTAS Science Imaging Instruments; Göttingen, Germany). Subsequently, 10,000 HeLa, 10,000 CaSki and 10,000 T98G cells were spiked into bone marrow samples, achieving a final concentration of 30,000 cell culture cells per 1 million bone marrow cells (ratio 1:33) and used as positive controls. MCF-7 cells were used separately as negative controls in a concentration of 200,000 cells per ml PBS. The cells were then centrifuged onto slides using a cytospin centrifuge, followed by fixation with ice-cold methanol for 5 min.

### 4.4. Sequential Immunofluorescence Staining and DTC Imaging

We have carefully modified a previously established sequential multi-parameter imaging process grounded in immunofluorescence staining techniques [23,24]. This enabled us to analyze Pan-CK, as an epithelial marker, Vimentin for DTCs with a mesenchymal phenotype, p16 as an HPV-associated marker, as well as PD-L1 and VEGF as potential indicators for prognosis and therapy, all within a single DTC. Additionally, to exclude hematopoietic cells, a counterstaining was performed using the leukocyte marker CD45 and DNA in cell nuclei was stained using DAPI.

Per patient, two slides containing 2 × 10^6^ prepared bone marrow cells were evaluated through this innovative multi-parameter staining process that employed antibodies with releasable fluorochrome conjugates. The use of releasing enzymes made the repeated staining of individual DTCs with up to six distinct markers possible. The Axioscan 7 scanning microscope (Carl Zeiss Microscopy, Jena, Germany) and Zeiss Zen 3.7 software enabled the annotation of individual cells and the precise documentation of their positions. To minimize imaging bias caused by autofluorescence or nonspecific background fluorescence, all immunofluorescence images were acquired using standardized imaging settings and identical exposure conditions for all samples. Candidate cells were not classified based on fluorescence signals alone, but additionally required distinct cellular morphology with an increased nucleus-to-cytoplasm ratio, positive nuclear DAPI staining, absence of CD45 expression, and positivity for at least one tumor-associated marker.

In analogy to Eckardt et al. [24], the protocol includes two rounds of staining and was performed as follows: starting with a rinse of the slides in TBS-T for 10 min at room temperature (RT), followed by a blocking phase to minimize cross-reactions for 30 min in 5% BSA in TBS-T at RT, and subsequent incubation with conjugated antibodies for 60 min at RT. In the first round of staining, anti-human Pan-Cytokeratin APC-conjugated REAdye_lease antibody (Cat. No. 130-123-091, Miltenyi Biotech, Bergisch Gladbach, Germany), anti-human vimentin FITC-conjugated REAdye_lease antibody (Cat. No. 130-127-022, Miltenyi Biotech, Bergisch Gladbach, Germany) and anti-human VEGF PE-conjugated REAdye_lease antibody (Cat. No. 130-118-061, Miltenyi Biotech, Bergisch Gladbach, Germany) were applied. Following antibody incubation, the excess staining solution was gently removed. This was followed by two washing steps in TBS-T for 10 min each at RT in the dark. Subsequently, the slides were rinsed twice with distilled water for 10 min each at RT, again in the dark. Any remaining droplets were carefully tapped off. Afterwards, a mounting medium enriched with DAPI for DNA nuclear staining (Slowfade Antifade Mounting Medium, Thermo Fisher Scientific, Waltham, MA, USA) was used before the initial imagery capture. The microscopic scans of the stained slides were acquired to identify the secondary fluorescence emitted from the fluorochromes using band-pass excitation filters D for DAPI (emission 465 nm), Y3 for phycoerythrin (PE; emission 561 nm) and Y5 for allophycocyanin (APC; emission 673 nm) at 20× magnification before uncovering the slides and releasing the antibody–fluorochrome complex from the cells using the REAlease Release Reagent (Cat. No. 130-120-675, Miltenyi Biotech, Bergisch Gladbach, Germany).

The release process was carried out as follows: Initially, the coverslip was carefully removed and the slides were rinsed with PBS for 10 min at RT. This was followed by incubation with the release reagent, prepared by mixing one part release reagent with 40 parts buffer, for 40 min in a dark, humidified chamber at RT. After incubation, the slides were rinsed with TBS-T for 10 min at RT.

In the second round of immunofluorescence staining, anti-human PD-L1 conjugated with AF 647 antibody (Cat. No. ab209960, abcam, Cambridge, UK), anti-human p16INK4a conjugated with AF488 antibody (Cat. No. ab199756, abcam, Cambridge, UK), and, for counterstaining, anti-human CD45 antibody conjugated with PE (Cat. No. 130-110-770, Miltenyi Biotech, Bergisch Gladbach, Germany) were applied, followed by image acquisition using the excitation filters mentioned above.

**Figure 9:**
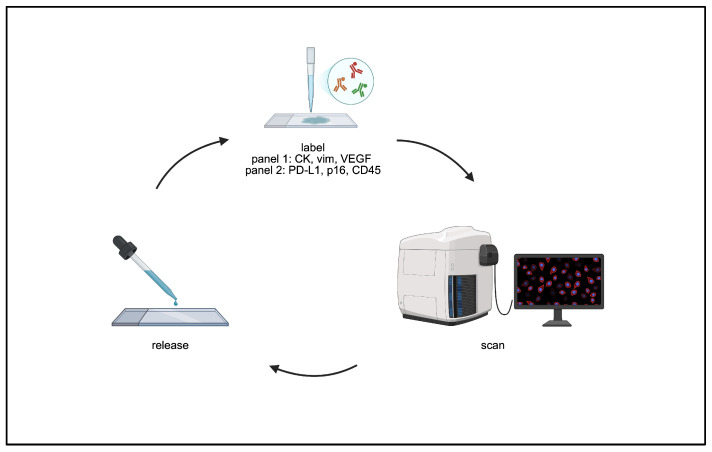
Sequential multi-parameter immunofluorescent staining. After applying the first antibody panel (CK, Vim and VEGF), the slides were scanned and DTC profiles were assessed. Subsequently, releasable fluorochrome-conjugates were digested and the slides were subjected to the second antibody panel (PD-L1, p16, CD45) followed by scanning and DTC detection. Created with BioRender.com.

For every patient sample, we acquired two whole slide images per channel (DAPI, PE, APC and FITC), resulting in four images per patient sample. For DTC analysis and quantification, the two resulting images of every patient sample were aligned via the Zeiss Zen software. Hence, using overlays of the two images of individual DTC candidates in each channel allowed the detection of distinct cellular profiles by positivity or negativity for CK, Vimentin, VEGF, PD-L1, p16INK4a and CD45 (Figure 9).

### 4.5. Release of Fluorochromes from APC-, FITC- and PE-Conjugated Antibodies for Further Staining

The REAlease-based fluorochrome removal strategy was applied as previously published by our group [24]. In this study, the protocol was extended by including an additional release step for PE-conjugated antibodies, enabling a three-channel release (allophycocyanin (APC), fluorescein isothiocyanate (FITC) and phycoerythrin (PE) instead of two channels, as described in the original protocol. To verify the efficiency of fluorochrome release, the analysis workflow described by Eckardt et al. [24] was applied. In brief, fluorescence intensities in cellular compartments of positive control cells were quantified before and after the release step using Zeiss Zen and Mathematica software (Version 13.3, Wolfram Research Inc., Champaign, IL, USA). As previously established, this included generation of Red Green Blue (RGB) cell masks, automatic thresholding using Otsu’s algorithm, and fluorescence quantification via integrated intensity and area coverage.

**Figure 10:**
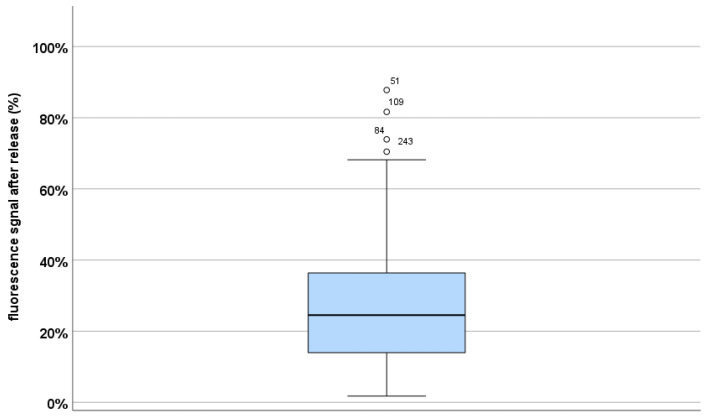
Box plot showing the residual fluorescence signal after the release step (%) in 295 CaSki cells following treatment with a VEGF-PE conjugated antibody. The release efficiency was evaluated by signal quantification in the cells before and after treatment with the release reagent. The median fluorescence signal was 24.5% (mean 26.7%), corresponding to a signal reduction of 75.5%. The range was 1.75% to 87.78%. Statistical graph was generated using IBM SPSS Statistics Version 29.0.

**Figure 11:**
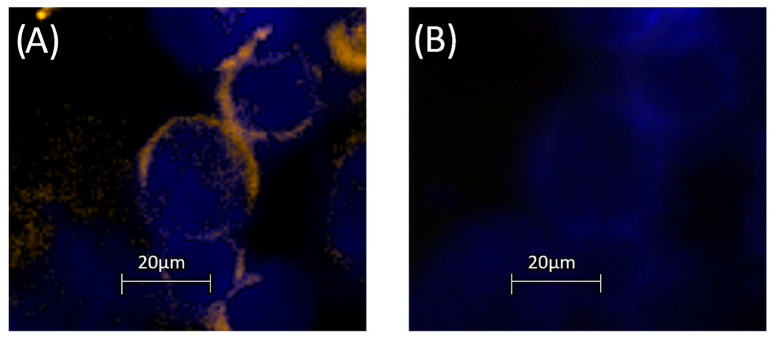
CaSki cells before and after the release step. Representative immunofluorescence image of two CaSki cells spiked into bone marrow cells (**A**) before and (**B**) after the release step. The PE channel shows VEGF staining before staining with CD45, while nuclei were stained with DAPI (blue). Images were processed using Zeiss ZEN software and figure was assembled using Microsoft PowerPoint.

In addition to the originally described APC and FITC channels, this study included the PE channel as a third released fluorochrome. The release efficiency for PE was evaluated analogously and revealed a reduction in dye intensity of 75.5%, comparable to the other channels. In total, we analyzed 295 CaSki cells that stained positive for VEGF-PE (orange). Results are summarized in Figure 10. Because of persisting autofluorescence in the bone marrow samples, signal reduction was slightly lower in the PE- compared to APC- and FITC-channel (PE 75.5%; FITC 75.8%; APC 77.3%). Nevertheless, the background signal was also detected in the negative control samples and was considered during interpretation of single-cell fluorescence signals. The achieved release efficiency was sufficient for practical application of the staining protocol, since the residual fluorescence signal was no longer detectable by visual inspection during image analysis as shown in Figure 11. In the second round of staining the PE channel was reused for detection of leukocyte marker CD45. In addition to residual background fluorescence that is not detectable by visual inspection, CD45-positive cells displayed a different expression pattern compared with the previously detected PE signal, allowing reliable discrimination between staining rounds. Cells with ambiguous residual fluorescence signals lacking distinct cellular morphology were excluded from further analysis. Since CD45 was used exclusively as an exclusion marker, residual PE fluorescence would more likely contribute to false-negative rather than false-positive DTC classification.

### 4.6. Immunohistochemical Staining of Matching Tumor Tissue

For our study, we performed re-analysis of p16, VEGF and PD-L1 expression of DTC-positive patients using formalin-fixed, paraffin-embedded (FFPE) specimens from tumor tissue. As mentioned above, the material was obtained by core needle biopsy (CNB) at primary diagnosis, at the time of recurrence or during primary oncological surgery. Immunohistochemical examination was performed in our Department of Pathology according to the ASCO/CAP guidelines using the Ventana-platform. PD-L1, p16 and VEGF staining results were reevaluated for this study [42,43,44,45].

### 4.7. Statistical Analysis

Descriptive statistics and Spearman Rho rank correlation were used to investigate associations between DTC subpopulations and clinical characteristics of the patients. We categorized DTC subpopulations into different groups such as CK-positive and CK-negative to find correlations between cellular markers like Vimentin and PD-L1. Clinical data were divided according to characteristics such as primary or recurrent disease. Mann–Whitney U and Chi-Square test were applied to analyze differences in the patients’ DTC profiles. The SPSS (IBM SPSS Statistics, Version 29.0) program was used, and statistical significance was set at *p* < 0.05.

## Figures and Tables

**Table 1:** Reference cells.

Cells/Marker	HeLa	T98G	CaSki	MCF7	Hematopoietic Cells
Pan-CK	positive	negative	positive	positive	negative
Vim	positive	positive	positive	negative	positive
VEGF	positive	negative	positive	negative	positive
PD-L1	negative	negative	positive	negative	both *
p16INK4A	positive	negative	positive	negative	positive
CD45	negative	negative	negative	negative	positive
DAPI	positive	positive	positive	positive	positive

Immunofluorescence (IF) staining patterns of the various markers on the cell lines HeLa, T98G, CaSki and MCF7 and hematopoietic bone marrow cells. * The majority of cells were negative, while activated B-and T-cells can express PD-L1.

**Table 2:** DTC numbers and characteristics among the patient cohort detected with the multi-parameter approach.

Pat. ID	Total DTCs	CK+ Count	CK− Count	Vim+ Count	Only Vim+ Count	CK+Vim+ Count	VEGF+ Count	PD-L1+ Count	p16+ Count
1266	15	10	5	8	2	3	2	2	4
1294	10	2	8	9	1	2	6	4	7
1362	5	3	2	3	1	2	2	1	0
1446	12	2	10	12	9	2	4	0	3
1481	4	0	4	4	4	0	0	0	0
1491	12	7	5	12	0	7	12	3	12
1707	2	0	2	2	1	0	1	0	0
1791	7	1	6	6	6	0	0	0	0
1911	8	0	8	8	8	0	0	0	0
1923	7	1	6	7	5	1	0	0	1
2018	9	2	7	7	5	1	2	2	2
2063	3	0	3	2	2	0	0	1	0
2223	7	0	7	7	2	0	5	1	4
2467	17	3	14	16	16	2	2	0	1
2562	3	0	3	3	2	0	0	1	0
2603	16	7	9	13	6	4	4	1	6
2683	11	5	6	8	4	2	1	2	2
2837	15	3	12	15	10	3	2	2	0
2931	5	3	2	2	2	0	0	0	0
2945	8	0	8	8	7	0	0	0	1
2982	6	2	4	5	4	1	1	0	0
3028	10	2	8	10	7	2	1	1	0
3031	1	0	1	1	1	0	0	0	0
3073	5	1	4	3	1	0	0	3	3
3377	14	4	10	11	10	1	0	0	1
3413	1	1	0	0	0	0	0	0	0
2799	1	0	1	1	0	0	1	0	1
3072	10	2	8	9	6	1	0	1	2
3166	6	2	4	3	3	0	0	0	1
3172	8	0	8	8	8	0	0	0	0
3282	5	2	3	4	3	1	1	0	0
3368	5	3	2	4	2	2	1	1	1
sum	248	68	180	211	138	37	48	26	52
mean	7.8	2.1	5.6	6.6	4.3	1.2	1.5	0.8	1.6
median	7	2	5.5	7	3.5	1	1	0	1

**Table 3:** Quantification of DTCs using multi-parameter IF staining method compared to the conventional immunocytochemical CK based detection.

Pat. ID	Total DTCs IF	CK+ Count IF	CK− Count IF	Total CK+ ICC
1266	15	10	5	9
1294	10	2	8	14
1362	5	3	2	20
1446	12	2	10	0
1481	4	0	4	0
1491	12	7	5	3
1617	0	0	0	0
1707	2	0	2	0
1740	0	0	0	0
1791	7	1	6	0
1800	0	0	0	0
1860	0	0	0	0
1911	8	0	8	1
1923	7	1	6	0
1951	0	0	0	0
2018	9	2	7	4
2063	3	0	3	0
2223	7	0	7	4
2467	17	3	14	2
2509	0	0	0	0
2562	3	0	3	0
2603	16	7	9	5
2683	11	5	6	30
2802	0	0	0	0
2837	15	3	12	18
2931	5	3	2	1
2945	8	0	8	2
2982	6	2	4	6
3028	10	2	8	31
3031	1	0	1	5
3060	0	0	0	0
3073	5	1	4	9
3377	14	4	10	4
3398	0	0	0	0
3413	1	1	0	3
1794	0	0	0	0
2107	0	0	0	0
2799	1	0	1	0
3072	10	2	8	11
3166	6	2	4	6
3172	8	0	8	1
3282	5	2	3	3
3368	5	3	2	2
sum	248	68	180	194
mean	5.8	1.6	4.2	4.5
median	5	1	4	1

**Table 4:** Patient characteristics.

	*n* = 43	%
Age		
Median (years)	45	
Range	30–73	
Menopausal status		
pre-	28	65.1
post-	14	32.6
Missing	1	2.3
Tumor stage		
T1	29	67.4
T2	8	18.6
T3	4	9.3
T4	1	2.3
missing	1	2.3
Nodal status		
Negative	35	81.4
Positive	7	16.3
Missing	1	2.3
Grading		
G1	7	16.3
G2	19	44.2
G3	16	37.2
Missing	1	2.3
Histological subtypes		
Squamous cell carcinoma	33	76.7
Adenocarcinoma	9	20.9
Adenoid basal carcinoma	1	2.3
Lymphovascular Invasion		
Absent (L0)	22	51.2
Present (L1)	19	44.2
Missing	2	4.7
Distant metastases		
No (M0)	41	95.3
Yes (M1)	2	4.7
Setting		
Primary Tumor	40	93
Recurrence at baseline	3	7
Treatment		
Surgery	32	74.4
Definitive CRT	8	18.6
NACT followed by surgery	1	2.3
Surgery followed by adjuvant CT	1	2.3
Surgery followed by CRT	1	2.3
Outcome		
Disease free	39	90.7
Recurrence during follow up	4	9.3

CRT: chemoradiotherapy; NACT: neoadjuvant chemotherapy; CT: chemotherapy.

## Data Availability

The original contributions presented in this study are included in the article. Further inquiries can be directed to the corresponding author.

## Abbreviations

The following abbreviations are used in this manuscript:

AF488	Alexa Fluor 488 (fluorescent dye)
AF647	Alexa Fluor 647 (fluorescent dye)
APC	allophycocyanin
ATCC	American Type Culture Collection
BSA	Bovine Serum Albumin
CaSki	Human cervical squamous cell carcinoma cell line
CD45	Cluster of Differentiation 45 (pan-leukocyte marker)
CNB	Core Needle Biopsy
CRT	Chemoradiotherapy
CTCs	Circulating Tumor Cells
DAPI	4′,6-Diamidino-2-Phenylindole (nuclear stain)
DMEM	Dulbecco’s Modified Eagle Medium
DTCs	Disseminated Tumor Cells
EDTA	Ethylenediaminetetraacetic acid
EMT	Epithelial–Mesenchymal Transition
FFPE	Formalin-Fixed Paraffin-Embedded (tissue preservation method)
FITC	Fluorescein Isothiocyanate (fluorescent dye)
HCT116	Human colorectal carcinoma cell line
HeLa	Human cervical adenocarcinoma cell line
HPV	Human Papillomavirus
IF	Immunofluorescence
ICC	Immunocytochemistry
MCF-7	Human breast adenocarcinoma cell line
MRD	Minimal Residual Disease
NACT	Neoadjuvant Chemotherapy
PBS	Phosphate-Buffered Saline
PD-L1	Programmed Death-Ligand 1
PE	Phycoerythrin (fluorescent dye)
p16	Cyclin-Dependent Kinase Inhibitor 2A (p16INK4a)
RGB	Red, Green, Blue
RPMI 1640	Roswell Park Memorial Institute medium 1640
RT	room temperature
T98G	Human glioblastoma cell line
TBS-T	Tris-Buffered Saline with Tween-20
VEGF	Vascular Endothelial Growth Factor
Vim	Vimentin
